# A qualitative study on the breastfeeding experience of mothers of preterm infants in the first 12 months after birth

**DOI:** 10.1186/s13006-019-0229-6

**Published:** 2019-08-01

**Authors:** Lina Palmér, Jenny Ericson

**Affiliations:** 1 Faculty of Caring Science, Work Life and Social Welfare, University of Borås, Borås, Sweden; 2 School of Education, Health and Social Studies, Dalarna University, Falun, Sweden; 3 Center for Clinical Research Dalarna, Uppsala University, Falun, Sweden; 4 Department of Pediatrics, Falu Hospital, Falun, Sweden

**Keywords:** Breastfeeding, Experiences, First year, Mothers, Preterm infant, Qualitative

## Abstract

**Background:**

Being a mother of a preterm infant (< 37 gestational weeks) puts the mother in a vulnerable and fragile situation wherein breastfeeding is an important part of becoming a mother and bonding with the infant. Nevertheless, the breastfeeding experience of mothers during the first year after a preterm birth has not been well studied. To develop professional caring and supporting relationships, it is important to address this knowledge gap. The aim of this study was to describe the breastfeeding experience of mothers of preterm infants from birth up until 12 months after birth.

**Methods:**

The data in this qualitative study are derived from a multicentre randomized controlled trial where 270 mothers of preterm infants provided 496 written comments through questionnaires containing open-ended questions. The questionnaires were sent to the mother three times during the first 12 months after birth. A thematic network analysis based on hermeneutical philosophy was used to analyse and interpret the resulting data to describe the mothers’ experiences of breastfeeding.

**Results:**

Three organizing themes, namely, *“navigating smoothly,” “navigating with a struggle”* and *“navigating in ambiguity”* were revealed in the mothers’ narratives regarding their breastfeeding experiences during the first 12 months after birth. These organizing themes were further interpreted as one global theme that was deemed “*A journey to finding one’s unique way in breastfeeding*.”

**Conclusion:**

Mothers of preterm infants are in an exposed and vulnerable situation when initiating breastfeeding during the first year. This situation leads to a unique journey wherein each mother navigates through breastfeeding depending on her individual situation. An awareness of the diversity of breastfeeding experiences may contribute to the provision of professional caring and supportive relationships.

**Trial registration:**

www.clinicaltrial.gov NCT01806480 registered 7 March 2013.

## Background

Mothers of preterm infants breastfeed to a lesser extent than mothers of full-term infants [1], which may be due to infant immaturity and a lack of support [2, 3]. Previous studies described that mothers of preterm infants are in a vulnerable situation, and that breastfeeding is an important part of becoming a new mother and bonding with the infant [4–6]. Due to this vulnerable situation, breastfeeding support is essential. However, professional breastfeeding support has been shown to vary widely for mothers of preterm infants due to the individual style of various healthcare professionals; support was shown to be either constructive or destructive [7]. This inconsistency puts the mother in a situation wherein she is exposed to different support styles that are not always sensitive to her unique situation [7]. This phenomenon is combined with the fact that preterm infants are immature in their breastfeeding behaviour and may require a long time to mature, which may be challenging and stressful for the mother. The challenges that may occur include, for example, infant sleepiness, vague feeding cues, latching difficulties and weak sucking [2, 3, 6]. Maternal challenges involved with breastfeeding a preterm infant may include feelings of guilt and failure, an insufficient milk supply and milk expression. Nevertheless, breastfeeding a preterm infant may also rebuild a connection and confidence with motherhood; therefore, breastfeeding can be a healing and a bonding experience [3, 6, 8].

Previous studies have described maternal experiences of breastfeeding a preterm infant during a stay at the neonatal unit and/or a few weeks after discharge. However, very few studies have described mothers’ experiences of breastfeeding during the first 12 months after the birth of a preterm infant [9]. Human experience is complex and cannot be understood by analysing parts or measuring aspects of breastfeeding, as we are also affected by social context. It is important to examine each mother’s experience of breastfeeding their preterm infant because each individual mother has the most knowledge about her own experience. Illuminating breastfeeding experiences may help health professionals to provide caring and supportive relationships through learning about the mothers’ individual experiences. Thus, the aim of this study was to describe mothers’ experiences of breastfeeding their preterm infants from birth up until 12 months after birth.

## Methods

During a randomized controlled trial (RCT), breastfeeding mothers of preterm infants (gestational age < 37 weeks) provided qualitative data in written form about their experiences of breastfeeding their preterm infants during the first 12 months after birth. The RCT aimed to evaluate a proactive breastfeeding support intervention and included 493 mothers of preterm infants. The study was conducted after discharge from six neonatal units in Sweden. The results from this RCT are presented elsewhere [7, 10–12].

In this study, exclusive breastfeeding was defined as feeding with breast milk only regardless of feeding method, but could include medications, fortification and vitamins. Partial breastfeeding was defined as feeding with breast milk in combination with formula and/or solid food. No breastfeeding was defined as full formula feeding and/or solid food with no breast milk intake. All the infants in this study were breastfeeding directly at the breast with the exception of one infant who was exclusively fed breast milk from a bottle. The characteristics of the participating mothers are presented in Table 1. The data consisted of 496 written comments received from 270 mothers from follow-up questionnaires that were sent to the mothers 8 weeks (8w) after discharge from the neonatal unit, and 6 (6 m) and 12 months (12 m) after birth between March 2013 and December 2015, as a part of the RCT. The follow-up questionnaire consisted of the following open-ended question regarding breastfeeding and the feeding experiences of the mothers: “*If you want, feel free to write about what you have experienced while breastfeeding/bottle-feeding your baby.”* The written narratives were combined in a Microsoft Word document.

**Table 1:** Characteristics of participants (*n* = 270)

Demographic variables	n (%) median [IQR] mean ± SD
Maternal variables	
Age, years	30.5 ± 4.8
Maternal educational level	
Higher education	154 (51)
Upper secondary school or less	116 (49)
Primipara	159 (59)
Mothers not born in Sweden	16 (6)
Vaginal birth	152 (56)
Multiple birth	32 (12)
Gestational age at birth, weeks	34 [2]
Exclusive breastfeeding	
at discharge	230 (85)
8 weeks after discharge	171 (63)
6 months after birth	75 (28)
Partial breastfeeding 12 months after birth	39 (15)

*SD* Standard deviation, *IQR* Interquartile range

### Analysis

The epistemological foundation for the analyses in this study was based on hermeneutical philosophy using a reflective lifeworld approach [13, 14]. A thematic network analysis, which aims to explore the understanding of an issue, was used to organize and interpret the data [15]. First, basic themes were derived from the text; basic themes support a statement or belief related to the diversity of the mothers’ experience, but they say very little by themselves. Then, these basic themes were interpreted into organizing themes in relation to the underlying narrative and meaning that they put forth. These organizing themes were more abstract and more revealing of the meaning within the texts. Finally, a global theme was deducted, and a comparative analysis among the organizing themes gave rise to the primary interpretation that linked all of the previous interpretations together into an overarching theme – the global theme, suggesting how to understand the phenomenon. During the analysis, rigor was maintained by trying to be both open and pliable as well as keeping a bridled attitude. More precisely, the researchers read the written text with an open mind until it felt familiar, after which the actual analysis was initiated. During the analysis, there was a movement between the whole (written text), the parts (basic themes) and the new whole (the global theme and the organizing themes). Movement during analysis and the methodological principles have guided the process of maintaining rigor. Quotes were used to support the interpretations of the text; the quotes that are presented in the results are labelled with each mother’s randomization code. After the analysis was finished and the quotes selected we added the infant’s gestational age (GA) at birth and infant feeding status in the previous 24 h at the last data collection point to each quotation to give a sense of each feeding path.

## Results

**Fig. 1:**
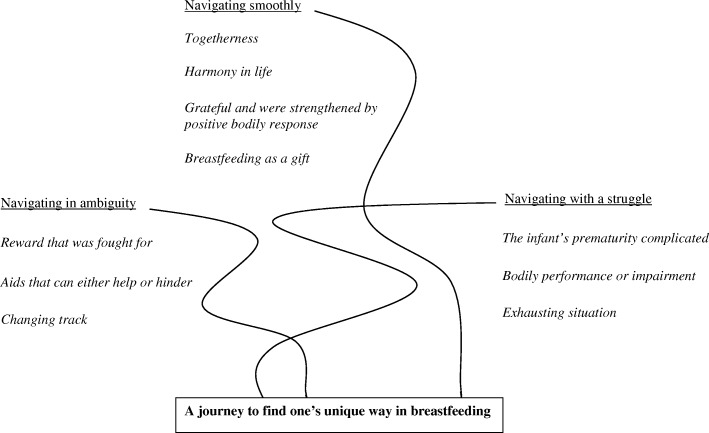
Basic themes, organizing themes and the global theme that illustrate the findings and the analytic process. The tracks are intertwined with each other and can be understood as tracks on a map where the goal is to find one’s own unique way in breastfeeding

The following three organizing themes were revealed in the mothers’ narratives regarding their breastfeeding experiences during the first 12 months after birth: *“navigating smoothly”*, *“navigating with a struggle”* and *“navigating in ambiguity.”* These organizing themes were summarized in one global theme: *“a journey to find one’s unique way in breastfeeding”* (Fig. 1).

### Navigating smoothly

Navigating smoothly through one’s breastfeeding journey means that breastfeeding was experienced in a positive way without any major problems or difficulties. In this theme, breastfeeding was a way to become close with the infant and thus strengthen the relationship between the mother and infant. The mothers experienced a feeling of togetherness with their infants. They described this feeling of togetherness as a mutual interaction and an intimate relationship with the infant. Such mutuality and intimacy was reported to provide a unique closeness and strong bond with the infant.

*It feels very good; breastfeeding provides great closeness and attachment, which gives me a great inner satisfaction as a parent. Now, after 6 months, I am so used to breastfeeding. In addition, my son eats more efficiently now, so it feels more practical to quickly and easily feed him compared with before, when you breastfeed all the time. SK5 (GA 36+2, breastfed exclusively at 6m)*

When breastfeeding was experienced as smooth, mothers felt a harmony in life when breastfeeding. Breastfeeding was described as a way to rest and calm down as well as a period of relaxation for both the mother and infant. In such harmonious breastfeeding situations, the mothers also felt a sense of wellbeing, tranquillity and security in breastfeeding.

*For me, breastfeeding is usually a quiet and harmonious moment - an opportunity to sit down and relax a little extra. SK59 (GA 34+3, breastfed exclusively at 6m and partially at 12m)*

Despite concerns about breastfeeding that some mothers experienced during pregnancy, it was a relief for many that everything worked well regarding breastfeeding. Breastfeeding seemed to be a smooth way of being with the infant and thus strengthened the mother’s confidence in parenting. This situation was characterized by simply being a breastfeeding mother without experiencing any problems or difficulties. Breastfeeding was integrated into the mother’s life in an embodied way.

*It feels very good to be able to breastfeed, and it works very well most of the time. SU38 (GA 35+5, breastfed exclusively at 8w and partially at 6m and 12m)*

Mothers also felt grateful and were strengthened by the positive bodily response of their breastfeeding. Pride in succeeding with breastfeeding was awakened, and breastfeeding was a privilege to experience. Happiness emerged because breastfeeding went smoothly and was pain free. Mothers also reported amazement over the body’s ability to produce enough breast milk for the infant to thrive, as there was often an inherent apprehension that this would not happen.

*Breastfeeding my child makes me proud; closeness and love grow between us. When I breastfeed her, we make eye contact and small talk, so I never get tired of breastfeeding her. I love breastfeeding her; I couldn’t replace this breastfeeding time with anything else. K45 (GA 31+0, breastfed exclusively at discharge and partially at 8w, 6m and 12m)*

Mothers were proud of their ability to manage breastfeeding and grateful for the closeness and growing love that breastfeeding provided. One mother of twins was proud that she managed to breastfeed her infants for 6 months, and she often breastfed the infants together. The positive breastfeeding experiences was etched in the mother’s memory after she ceased breastfeeding. Another mother wrote; *I felt happy and satisfied every time I nursed my son; it was a good feeling that I will not forget. K67 (GA 34 + 5, ceased breastfeeding at 5 months).*

Yet another mother wrote:

*To breastfeed my child is pure love. I get the chills in my body when I think about ceasing breastfeeding (in some years or so) because I like it. It is our moment. SK67 (GA 31+5, breastfed exclusively at 6m)*

The mothers also described breastfeeding as a gift to the infant. Breastfeeding gave the mothers a lovely feeling, and it was satisfying to provide the infant with nutrition and protection, which was perceived as being the best possible start. It was described as *“awesome”* and *“beautiful”* when the infant became satisfied by being breastfed.

*I give my child the best she can get nutritionally. She gets security and comfort when she lies at the breast. We have a nice emotional contact. Ö71 (GA 35+6, breastfed exclusively at 6m and partially at 12m)*

*It is a great gift to give to your child. It is convenient to always have the food with you. F23 (GA 34+0, ceased breastfeeding at 8 months)*

### Navigating with a struggle

Navigating with a struggle means that the breastfeeding journey is experienced more or less as a bodily performance instead of a smooth relationship with the infant. The most prominent problem or difficulty faced by the mothers was that the infants’ prematurity complicated breastfeeding. The mothers reported that their infants could not or did not want to breastfeed. The infants could have a weak suck or did not suck. The interaction and relationship between the mother and infant were, or could be, complicated. Sometimes, the mother wanted to breastfeed but the infant did not. Some mothers accepted this situation, but other mothers wished that they had tried harder or longer, or they wished for more support. This failure of acceptance sometimes led to sadness and regret over the loss of breastfeeding, which was emotionally difficult to handle.

*It has been tough when my daughter just fell asleep at the breast. She has not received the whole meal at the breast. My milk dried up when she did not suck hard enough. (I) express milk and feed by bottle, and the feedings have taken a lot of time and effort. Ö19 (GA 32+2, ceased breastfeeding at 11 months)*

The struggle could also be over one’s own bodily performance or impairment. Mastitis, breast pain and/or a low milk supply (both perceived and actual) or maternal illnesses can disrupt breastfeeding, which can lead to stress, anxiety and frustration.

*I had pain from one breast during breastfeeding ever since he was small. I had candida in the milk ducts and sore nipples in the first 3 months, so it has been difficult, but I do not want to stop breastfeeding. Ö60 (GA 36+2, ceased breastfeeding at 9 months)*

Struggling with breastfeeding may also be associated with having too much milk and thus being confronted with extreme bodily changes in the breasts. Such bodily changes in the breast may in some cases be experienced as very trying and, in some cases, these changes of the breast can be perceived as disgusting.

Breastfeeding is sometimes seen by health professionals as only food, and this mind set can be transferred to the mothers. This attitude places a huge focus on breastfeeding as food, infant weight gain, and breast milk production, which can create feelings of breastfeeding as an accomplishment or, as one mother wrote: *maternal and infant eating disorders. Ö20 (GA 31 + 4, ceased breastfeeding at 5 months).*

Another mother described a similar experience:

*Now, afterwards, I see that initially my child was “just” weight gain. Everything was about gaining weight, and the only thing I saw was the child's needs (nutritional needs) - not the CHILD, not the interaction through movements and smiles, just weight. It (breastfeeding) became “mechanical” - food - weight. No one saw my little child. F99 (GA 33+0, partially breastfed 8w)*

Breastfeeding was sometimes seen as an exhausting situation that caused the mothers to experience stress. Furthermore, feelings of failure, of being solely responsible, being insufficient or incapable were also expressed. These feelings sometimes led to disappointment and frustration that breastfeeding did not work out as expected. Breastfeeding was reported by some mothers to be mentally tough and unpredictable; moreover, the need to always be close to the infant and the inability to leave was tiring for some mothers. Some mothers wanted to share infant feeding with the father, and some did so.

*The worst thing I have experienced. I felt really bad due to all the stress and pressure about breastfeeding and the idea that it (breastfeeding) would be “the best” option. Bottle-feeding is a pure dream in comparison. Breastfeeding is not free; it is the most costly thing I have experienced. I lost myself completely. SU68 (GA 33+3, partially breastfed 8w)*

### Navigating in ambiguity

Navigating in ambiguity means that the breastfeeding journey could be seen as a reward that was fought for. The initiation of breastfeeding, and occasionally later during the first year, could be tough and difficult. However, after a short or long (days to months) struggle, mothers overcame the difficulties, and breastfeeding became well-functioning and often pleasurable.

*I had problems with breastfeeding in the beginning and felt that it (breastfeeding) started to be associated with anxiety. After about 3 months, things turned around, and now I love to breastfeed. SU23 (GA 36+4, breastfed partially at 8w and exclusively at 6m)*

Expressing breast milk or using nipple shields and bottles are aids that can either help or hinder breastfeeding. Mothers described milk expression as frustrating and difficult. However, they also reported pride in having expressed milk for their infants when they later managed to breastfeed their infants directly at the breast. Some mothers continued to express milk to provide the infant with their breast milk when the infants could not breastfeed directly at the breast.

*“It has been hard, sometimes painful, and it was necessary to express a lot of milk. When it (breastfeeding) worked well (most of the time), it has provided moments of closeness with my baby”. T40 (GA 33+0, ceased breastfeeding at 9 months)*

Nipple shields were described as either a saving solution or a hindrance. The nipple shield was experienced by some mothers as an aid that helped the infants to get a good latch. However, it was a relief when the infants started to breastfeed without the nipple shield. On the other hand, the nipple shield was also experienced by some mothers as bothersome, a failure and/or difficult to handle. The mothers who still used a nipple shield because the infants did not latch without it wished for the infant to manage breastfeeding without the nipple shield in order to be free and not bound by the shield.

*It (breastfeeding) is not as smooth as I thought from the beginning when I have used a nipple-shield because my child is unable to suck otherwise. T20 (GA 33+1, ceased breastfeeding at 7 months)*

Bottles were reported by some mothers to interfere with breastfeeding. These mothers reported that after introducing bottles, the infant preferred the bottle over the breast, which led to the cessation of breastfeeding. Bottles and formula feeding were, in some situations, also described as a rescue when breastfeeding had been tough or did not work. The switch to bottle-feeding facilitated maternal well-being in some cases.

*Breastfeeding didn't suit me, and it was the best decision for me and my family to start giving formula. I felt bad when breastfeeding. SU61 (GA 32+6, fully formula fed at 8w)*

When navigating breastfeeding with ambiguity, changing track was one way to find one’s own way in the breastfeeding situation. Changing track means that breastfeeding was initiated but, after discovering an unwillingness, not feeling well or feeling uncomfortable, the mothers ceased breastfeeding. Feeling “disgusting” or “like a cow” was also reported. For these mothers, breastfeeding felt wrong, they ceased breastfeeding and even regretted that they had started to breastfeed.

*I thought I would be a mom who would like to breastfeed, but the opposite turned out to be true. F11 (GA 35+6, breastfed exclusively at 8w and had ceased before 6m)*

### A journey to find one’s unique way in breastfeeding

The three organizing themes were used to construe an overarching interpretation, a global theme. The interpretations suggested that the mothers’ breastfeeding experiences could be described as a journey to find one’s unique way in breastfeeding when in the vulnerable situation as a new mother with a preterm infant. The journey to become a breastfeeding mother began during pregnancy and was physically stopped when breastfeeding was ceased. However, breastfeeding did not entirely stop when breastfeeding ended physically, because the experience continued in the mother’s memory. The breastfeeding journey consists of navigating one’s own unique needs as a mother as well as the unique needs of the infant. Such navigations of multiple, sometimes competing needs, are complex, which may challenge mothers. The mother thus navigates with both her own inner wishes and needs, as well as the perceived wishes and needs of the infants while, at the same time, navigating through prevailing norms and opinions, especially those given by health professionals. Three tracks of navigation were evident based on the data, namely, navigating smoothly, navigating with a struggle or navigating in ambiguity. These tracks were, to some extent, intertwined with each other and could be understood as tracks on a map where the goal was to find one’s own unique way (Fig. 1).

## Discussion

Our findings in the mothers’ narratives regarding breastfeeding experiences during the first 12 months after the birth of a preterm infant revealed three organizing themes, namely, *“navigating smoothly”, “navigating with a struggle”* and *“navigating in ambiguity”,* which were interpreted as one global theme: “*A journey to find one’s unique way in breastfeeding.*”

Breastfeeding as a journey has been described in the context of full-term infants, as an “engrossing, personal journey” which is physical and requires maternal commitment, adaptation, and support [16]. Breastfeeding has also been described as a personal choice, harder than expected and as being exposed to public debates [17]. In the context of preterm infants, the present study highlights breastfeeding as a journey in a slightly different way. One positive finding was that breastfeeding worked out well for many mothers; they enjoyed breastfeeding and the feelings and advantages that it entailed for both themselves and their infant. This finding is not usually described in research but is important to highlight, as it is a further reason to support breastfeeding. The present study also highlighted breastfeeding a preterm infant as a relational activity that the mother performs as part of mothering the infant. The mothers described many aspects of their experiences of breastfeeding their preterm infants; breastfeeding was not solely about nutrition and protection but also about combinations of many elements, such as emotions, love, caring, and relationships, which could be both positive and negative.

Mothers who were not able to fulfil their breastfeeding wishes or who discovered that they did not enjoy breastfeeding were an especially vulnerable group, both due to having a preterm infant and not being able to breastfeed as they had expected. This extra vulnerability must be considered when caring for the mother and infant; otherwise, the suffering of both the mother and infant can be overwhelming for the mother to handle. Recent research regarding breastfeeding difficulties showed that some mothers may feel lost in motherhood, feeling as though she has an insufficient body, as well as having difficulties establishing a relationship with the infant [18]. van Wijlen [19] suggests that breastfeeding needs to be seen as a relationship, and healthcare attitudes about breastfeeding must shift towards a relational approach instead of the disembodied and often mechanistic approach that often exists in the dominant Western medical model. Furthermore, according to a holistic approach to care, it is important to discuss the concept of the lived body (i.e. the human being cannot be separated from the world or into a separate body and mind) [20]. Human beings must be seen as whole entities wherein the body-mind-world are integrated into the lived body. According to this, it is not always helpful to simply tell a mother who wants to breastfeed but cannot due to difficulties that “you are a good mother even if you cannot breastfeed”. This is because her breastfeeding experiences are intertwined with her view of being a mother. A caring attitude requires openness and a willingness to encounter each mother’s life situation, to create an environment in which mothers feel their perspectives are genuinely heard, valued and respected [21]. This approach requires that healthcare practitioners genuinely listen to what the patient expresses, both verbally and with body language [22, 23]. Hence, breastfeeding support should be given according to the individual mother’s unique wishes and needs regarding mothering and breastfeeding, as well as to establish a close and protective relationship with the infant. Furthermore, research [24–27] also suggests that breastfeeding should be promoted as a woman’s right and a feminist issue and not only as a women’s duty or responsibility due to the biological body and the physical health benefits of breastfeeding. However, social and structural inequities may influence a woman’s ability to choose to breastfeed [28], which is important to be aware of when supporting breastfeeding. Altogether, it is important to approach breastfeeding as a relation with the mother’s experiences and life situation in mind.

As previously mentioned, the breastfeeding journey can vary greatly among mothers, and breastfeeding a preterm infant may be a multifaceted and complex experience. In a study by Niela Vilén et al., breastfeeding mothers of preterm infants were divided into different typologies [9]. This means that the mothers were categorized into typologies because the mothers in that study maintained their feelings, either positive or negative, during the follow-ups. There is a risk of categorizing mothers with preterm infants and considering them as a homogeneous group based on the infant’s prematurity. Our results show that breastfeeding experiences are more complex than that. Breastfeeding a preterm infant during the first year was not shown to be straightforward; rather, it was like following a winding path, going forward but sometimes backward or sideways. There may therefore exist a danger in classifying and categorizing mothers because doing so takes for granted that the present situation will also persist in the future. Our results show that there needs to be a recognition that the journey in breastfeeding takes different paths during the entire breastfeeding period.

Furthermore, when comparing other research about mothers’ experiences of breastfeeding their preterm infants, it seems that breastfeeding mothers of preterm infants in different Western countries experience certain similarities. A recurrent issue in the studies, including ours, was that the infant’s prematurity complicated breastfeeding [2, 9]. This issue could be addressed in neonatal care to identify care routines that support the preterm infant’s development as well as to educate parents about preterm infant feeding development and how to support the infant during breastfeeding to enable exclusive breastfeeding. In this study, with the exception of one mother, all mothers breastfed their preterm infants directly at the breast, which should be taken into account when interpreting the results. In some other countries, it is more common to feed infants expressed breast milk in a bottle for various reasons, including care routines, society norms and/or personal reasons [29, 30].

A strength of this study is the variety of descriptions during a long follow-up and the relatively representative sample of breastfeeding mothers of preterm infants in a Swedish context. One limitation may be that the comments provided by the mothers are, to some extent, short; however, these comments provide a rich variation in the descriptions of lived experiences of breastfeeding during the first year as a mother of a preterm infant. To gain an even deeper understanding of the possible changes and their influences during the breastfeeding journey, regular individual interviews during the breastfeeding period are warranted. The breastfeeding support given in the RCT in the first 2 weeks after discharge may have influenced the mothers’ experiences of breastfeeding. However, the support was provided for only a short period and not all mothers took advantage of the support offered. Hence, we do not believe it to have affected the experiences of breastfeeding over the 12 m period to any great extent. Another limitation of the study is that it only included mothers who were breastfeeding at discharge and did not include experiences of mothers who ceased breastfeeding while in the neonatal unit potentially impacting generalizability.

## Conclusion

Mothers experienced breastfeeding their preterm infants in different ways, and each mother found her own way in breastfeeding. An awareness of the diversity of breastfeeding experiences may help provide better professional caring and supporting relationships. The whole care chain for preterm infants and their mothers (e.g., maternity, neonatal and child health care) needs to have a caring approach and holistically meet the uniqueness in every mother’s breastfeeding situation. Such an approach enables health care professionals to prepare, care for and support the breastfeeding mothers of preterm infants, to encounter each mother where she is and to give each mother the right to her own breastfeeding journey.

## Data Availability

The dataset used and analysed during the current study are available from the corresponding author on reasonable request.
